# Cancer-Associated Fibroblasts in Gastrointestinal Cancers: Unveiling Their Dynamic Roles in the Tumor Microenvironment

**DOI:** 10.3390/ijms242216505

**Published:** 2023-11-19

**Authors:** Noor N. Al-Bzour, Ayah N. Al-Bzour, Obada E. Ababneh, Moayad M. Al-Jezawi, Azhar Saeed, Anwaar Saeed

**Affiliations:** 1 Department of Medicine, Division of Hematology & Oncology, University of Pittsburgh Medical Center (UPMC), Pittsburgh, PA 15232, USA; nnalbzour211@med.just.edu.jo (N.N.A.-B.); analbzour20@med.just.edu.jo (A.N.A.-B.); 2 Faculty of Medicine, Jordan University of Science and Technology, Irbid 22110, Jordan; oeababneh185@med.just.edu.jo (O.E.A.); mmaljezawei19@med.just.edu.jo (M.M.A.-J.); 3 Department of Pathology and Laboratory Medicine, University of Vermont Medical Center, Burlington, VT 05401, USA; azsaeed29@gmail.com; 4 UPMC Hillman Cancer Center, Pittsburgh, PA 15232, USA

**Keywords:** cancer-associated fibroblasts, gastrointestinal malignancies, single-cell analysis, immune system

## Abstract

Gastrointestinal cancers are highly aggressive malignancies with significant mortality rates. Recent research emphasizes the critical role of the tumor microenvironment (TME) in these cancers, which includes cancer-associated fibroblasts (CAFs), a key component of the TME that have diverse origins, including fibroblasts, mesenchymal stem cells, and endothelial cells. Several markers, such as *α-SMA* and *FAP*, have been identified to label CAFs, and some specific markers may serve as potential therapeutic targets. In this review article, we summarize the literature on the multifaceted role of CAFs in tumor progression, including their effects on angiogenesis, immune suppression, invasion, and metastasis. In addition, we highlight the use of single-cell transcriptomics to understand CAF heterogeneity and their interactions within the TME. Moreover, we discuss the dynamic interplay between CAFs and the immune system, which contributes to immunosuppression in the TME, and the potential for CAF-targeted therapies and combination approaches with immunotherapy to improve cancer treatment outcomes.

## 1. Introduction

Gastrointestinal (GI) cancers, including esophageal, gastric, liver, pancreatic, and colorectal cancers, constitute a group of highly aggressive malignancies with significant cancer-related mortalities [1]. In recent years, research has unveiled the pivotal role of the tumor microenvironment (TME) in the initiation and progression of these cancers. The TME is a complex network of non-cancerous host cells, such as fibroblasts, endothelial cells, neurons, adipocytes, adaptive and innate immune cells, as well as non-cellular components like the extracellular matrix (ECM), and various soluble products, including chemokines, cytokines, growth factors, and extracellular vesicles [2].

Among the diverse stromal cells within the TME, cancer-associated fibroblasts (CAFs) have emerged as they resemble the main compartment of host stromal cells. They exert significant tumor-modulating effects that include the extracellular matrix, blood vessels, the immune system, tumor metabolism, and drug resistance [3,4].

Recent studies have unveiled intriguing findings regarding CAFs, particularly their role in gastrointestinal cancer metastasis. Notably, a secreted protein called SLIT 2, originating from CAFs, has been found to promote gastric cancer metastasis by binding to its receptor Robo1 [5]. In addition, the knockdown of SLIT2 in glioblastoma has shown tumor suppression effects indicating its tumor-promoting effects [6]. However, it has been reported that SLIT2 has an inhibitory role in the progression of many tumors including pancreatic, lung, breast, and thyroid cancers [7,8,9,10,11].

In this comprehensive review, we aim to provide an updated understanding of the role of CAFs in GI cancer. We will emphasize the significance of CAF markers and subsets, exploring the latest insights obtained using single-cell analysis and spatial transcriptomics. Additionally, we will discuss the potential implications of CAFs on immunotherapeutic agents’ efficacy, aiming to shed light on promising therapeutic avenues.

## 2. The Origin of Cancer-Associated Fibroblasts

CAFs emerge as vital components within the intricate milieu of the tumor microenvironment, engaging in direct crosstalk with cancer cells and endothelial and inflammatory cells [3]. Evidence from clinical and experimental studies supports the foundational role of CAFs in promoting tumor progression [3]. Nevertheless, CAFs display intricate biological properties, marked by distinct functions. Notably, Rønnov et al. underscored the diverse origins of breast cancer CAFs, deriving from sources such as residual fibroblasts, vascular smooth muscle cells, and pericytes, highlighting their lineage diversity [3]. Moreover, a spectrum of potential sources is evident from in vivo and in vitro studies, encompassing local infiltrating fibroblasts, endothelial cells, pericytes, adventitial fibroblasts, and cancer cells undergoing fibroblastic transformation, contributing to the diverse attributes of CAFs [3].

Importantly, a bone marrow transplantation model demonstrated the potential trans-differentiation of bone marrow cells into CAFs within the cancer microenvironment, suggesting an immigrant cell population as a source, thereby perpetuating the recruitment of CAF progenitors into cancer tissue and reshaping stromal reactions like fibrosis [3].

The historical foundation of fibroblasts, as elucidated by Virchow and Duvall, characterizes these cells as collagen-synthesizing entities within connective tissues. Normally present as single cells in the interstitial space and near capillaries, fibroblasts are integral to the diverse connective tissue matrix, often resembling mesenchymal stem cell precursors [12].

CAFs trace their origins to variable sources, including tissue-residual fibroblasts such as pancreatic stellate cells and hepatic stellate cells (HSCs), mesenchymal stem cells (MSCs) from normal tissues, endothelial cells undergoing endothelial-to-mesenchymal transition (EndMT), and cancer cells undergoing epithelial-to-mesenchymal transition (EMT) [13]. This complexity is exemplified in hepatocellular carcinoma (HCC), where CAF origins exhibit heterogeneous distribution, mainly observed in the tumor fibrous septum, fibrous capsule, and hepatic blood sinusoids [13]. The presence of α-smooth muscle actin (α-SMA)-positive HCC cells near blood sinusoids suggests origins from HCC cells undergoing EMT, linking CAFs to migration and invasion [13]. Under hypoxic conditions, heightened Fibroblast Activating Protein (FAP) gene expression and a classical EMT phenotype emerge in HCC cells, shedding light on CAF origin and influence within the HCC microenvironment [13].

Several markers have been identified to label CAFs, such as α-smooth muscle actin (α-SMA), fibroblasts activation protein (FAP), fibroblast-specific protein-1 (FSP-1), platelet-derived growth factor receptor (PDGF-R), and podoplanin [14]; however, these are not expressed on CAFs exclusively. The recent identification of protein markers that are CAF-specific and showed a correlation with prognosis includes collagen 11-α1 (COL11A1) [15] and microfibrillar-associated protein 5 (MFAP5) [16]. Tenascin-C (TNC) is an extracellular matrix glycoprotein that interacts with CAFs and promotes epithelial-to-mesenchymal transition (EMT) by upregulating EMT-specific markers such as vimentin and Snail in many cancers, including colorectal cancer and hepatocellular carcinoma, thus leading to poor prognosis [17,18]. These markers may serve as candidates for therapeutic targets.

In summary, comprehending the diverse and intricate origins of cancer-associated fibroblasts (CAFs) is pivotal for deciphering their dynamic interactions within the tumor microenvironment of gastrointestinal cancers. These origins not only encompass various cellular sources but also unveil the multifaceted nature of CAFs’ roles in tumor progression and shaping the immune response [3,12,13]. As research advances, unraveling the intricate mechanisms driving CAF generation continues to be an intriguing and evolving area of study, holding promise for enhancing our understanding of their heterogeneous nature and functional diversity [3,12,13]. This understanding could potentially pave the way for innovative therapeutic strategies targeting CAFs and their impact on tumor-immune interactions.

## 3. The Multifaced Role of CAFs in the Tumor Microenvironment

Over the past decade, cancer has surfaced from being solely a disease centered on malignant cells, rather than being a condition marked by a fundamental imbalance in the entire cellular environment. Among this cellular environment are CAFs, which are the most abundant cellular infiltration in tumor stroma and the microenvironment, making them prominent targets in solid tumors [19]. Fibroblasts impose a significant impact on various stages of cancer development with their ability to change their characteristics and release a diverse array of signaling molecules within the tumor microenvironment (TME) [1].

CAFs do not form a homogenous cell population, and their impact can vary depending on the specific system, exhibiting either tumor-promoting or tumor-suppressive effects [2,3]. The identification of CAF subtypes remains challenging due to their significant heterogeneity and phenotypic plasticity and the absence of well-defined markers [4,5]. This heterogeneity is largely attributed to their cellular origin, phenotype, and function. The expression of several Micro-RNAs (miRNA) and chemokine and cytokine signals can play a role in the transformation of normal fibroblasts into CAFs in gastric and ovarian cancers [20].

### 3.1. CAFs as Drivers of Tumor Progression

As many subpopulations of CAFs exist in the TME, they exhibit different roles in tumor growth, in which some subsets act as promoters of tumor progression while other subsets act as repressors [21,22]. The CAFs that participate in tumor progression participate in tumor developmental stages in a variety of pathways (1). Tumor progression can be facilitated by CAFs through their interaction with cells that infiltrate the tumor such as tumor-associated macrophages (TAMs), tumor-associated neutrophils (TANs), and tumor-associated mast cells [23,24,25]. Other CAF’s tumor-promoting effects include activating tumor angiogenesis, immune suppression, invasion of cancer cells, and metastasis. CAFs interact with these cells and foster polarization and promotion of their malignant transformation, which ultimately results in suppressing the immune response in the TME [25,26] (2).

Moreover, CAFs modulate tumor progression by remodeling the extracellular matrix (ECM), specifically by altering the ECM composition and integrity in the TME. These roles are guided by the ability of CAFs to secrete various growth factors and proinflammatory cytokines, such as transforming growth factor-β (TGF-β), vascular endothelial growth factor (VEGF), CXC-chemokine ligand (CXCL12), and interleukin-6 (IL-6) (3,4). In addition to their inherent immune-regulating capabilities, α-SMA+ CAFs could potentially disrupt the adaptive immune response through various mechanisms. TGF-β and IL-6 derived from α-SMA+ CAFs are linked to the inhibition of dendritic cell (DC) function and maturation. This, in turn, hampers T-cell activation, which promotes the induction of T-cell anergy (5). Precisely speaking, CAFs can facilitate the transformation of the ECM structure by secreting matrix metalloproteinases (MMPs) in addition to growth factors such as TGF-B1, which was reported to be the most important modulator in ECM remodeling (6–9). This process in turn yields a positive feedback loop in which the newly remodeled and transformed ECM exerts promoting effects on CAFs, thus maintaining their phenotypic features and enhancing tumor-promoting effects [27]. In addition, CAFs of endothelial or epithelial origin usually express FSP1/S100A4 and play a role in inducing tumor metastasis and immune evasion through chemokine ligand 2 (CCL2) [17,18].

Available evidence suggests that the remodeled ECM is associated with the invasion and metastasis of cancer cells [28,29]. This occurs when the ECM protein network that was remodeled by CAFs acts as a barrier against different immune cell populations, hence reducing their firing and response in the TME [30,31].

### 3.2. CAFs as Inhibitors of Tumor Progression

Several markers expressed in CAFs have been associated with anti-tumor effects and good prognosis. Podoplanin (PDPN)-positive CAFs were reported to be associated with good survival outcomes in small-cell lung cancer as these cells can inhibit tumor proliferation. However, PDPN-positive CAFs were associated with worse survival outcomes in non-small cell lung cancer [32]. This indicates the different roles of CAFs across different tumors. Other CAF-related markers that have been associated with dual roles include membrane type 1–MMP (MT1-MMP), periostin (POSTN), and hyaluronic acid (HA) [33,34]. The dichotomous role of these cells as tumor-inhibitory or tumor-stimulatory is regulated and controlled by the TME in which they occur. Studies have shown that the ‘desmoplastic’ and ‘immune’ subtypes of CAFs have tumor-inhibitory properties. This could provide a new potential to improve patient care by developing therapeutic strategies that target the tumor-stimulating CAFs while reinforcing tumor-inhibitory CAFs [35].

## 4. Remodeling the TME of Primary Gastrointestinal Tumors

### 4.1. Pancreatic Adenocarcinoma

Pancreatic cancer presents unique challenges due to its immunosuppressive TME. CAFs have been found to play an essential role in pancreatic cancer desmoplastic TME through increased production of stromal components such as collagen and hyaluronic acid [36,37]. Öhlund et al. classified CAFs into two major subtypes: inflammatory CAFs (iCAFs) and myofibroblasts CAFs (myCAFs) [38]. iCAFs are characterized by low expression of α-SMA and ACTA2 and a high expression of IL-6, IL-11, leukemia inhibitory factor, and STAT3. myCAFs have the opposite expression of iCAFs with a higher α-SMA expression and a lower IL-6 expression. They also showed that treating these cells with AZD1480, a JAK inhibitor, can disproportionately inhibit iCAFs more than myCAFs. myCAFs are also associated with increased deposition of collagen, which contributes to both angiogenesis and hinders drug delivery to cancerous cells [12]. During collagen cross-linking, YAP secretion is increased from CAFs leading to a stiff matrix. Matrix stiffness in turn leads to elevated YAP activity in CAFs, resulting in more activation of CAFs and more matrix stiffening. Overall, this resembles a vicious cycle between CAFs and TME through YAP signaling. High YAP has been associated with poor prognosis in pancreatic cancer [39]. Metformin, a diabetes medication, has been found to activate AMPK, which in turn inhibits YAP, potentially breaking the aforementioned vicious cycle [40,41]. Metformin has been found to have a favorable prognostic effect in local and locally advanced pancreatic cancer patients [41]. Targeting IGF-1 and IGF-2, which are secreted by macrophages and myofibroblasts, has been found to sensitize pancreatic tumors to gemcitabine [42]. Li et al. found that radiotherapy can activate CAFs by inducing CXCL12 and promoting invasion and epithelial-to-mesenchymal transition (EMT) [43]. This might explain radiotherapy resistance and offer a new biomarker for targeted approaches before or concurrently with radiation-based treatments in solid tumors like pancreatic adenocarcinoma.

### 4.2. Gastric Cancer

The poor prognosis of gastric cancer patients is mainly attributed to metastasis [44]. IL-6 secreted by CAFs could promote EMT and GC cells by activating the JNK2/STAT3 signaling pathway, which also leads to metastatic disease [45]. Ham et al. found that IL-6 also plays a role in chemoresistance to 5-FU and cisplatin [44]. They also observed that using Tocilizumab, an anti-IL-6R, leads to regaining sensitivity to 5-FU. CAF’s overexpression of Galectin-1 has been found to contribute to EMT gastric cancer cell lines [46]. Also, Galectin-1 can induce gastric cancer angiogenesis [47]. HGF is one of the paracrine proteins highly secreted by CAFs [47]. This can bind with c-Met expressed on cancer cells inducing their proliferation. This way, high CAF cells can lead to resistance to c-Met inhibitors [47,48].

Another mechanism by which CAFs play a role in GC is through bacterial infection. The bacteria Helicobacter Pylori is associated with the development of GC. Several studies suggest that the H. Pylori infection alters the mRNA expression and induces the differentiation of fibroblasts into CAFs, as well as initiating epithelial-to-mesenchymal transmission (EMT), thereby promoting tumor progression [49]. This was supported by Xu et al.’s study, in which they reported an existing synergistic relationship between CAFs and endothelial cells, where the TGF-B secreted by CAFs acts as a paracrine factor in enhancing the efficacy of EMT [50,51]. Moreover, they found that B-cells antagonize CAF effects and hence, can be used to suppress tumor growth [51].

Another way by which the interaction between CAFs and H. Pylori infection affects tumor growth is reported in a study by Shen et al. The authors reported that H. Pylori promotes tumor progression by increasing vascular adhesion molecule 1 (VCAM1) in CAFs by activating the JAK/STAT1 signaling pathway, which can further induce tumor invasion by interacting with integrin αvβ1/5 [52].

### 4.3. Esophageal Cancer

High CAF infiltration is associated with poorer prognosis in esophageal squamous cell carcinoma [53,54]. Zhao et al. studied the role of exosomal sonic hedgehog (SHH) secreted by CAFs [55]. They found that it promotes the proliferation and migration of esophageal squamous cell carcinoma. Treating these cells with cyclopamine, an inhibitor of the SHH pathway, partially neutralized the effect of CAF-derived exosomes. Zhang et al. found that FGFR2 was universally upregulated in all CAF samples compared with normal fibroblasts. FGFR2 also induced cancer cell growth rapidly when cultured with CAFs compared with normal fibroblasts [56]. Using FGFR2 inhibitors is currently under clinical investigation in esophageal carcinoma [57]. Both HGF/MET and FGF/FGFR axes were found to induce resistance against lapatinib in esophageal squamous cell carcinoma [58]. Noma et al. reported that even with the presence of high levels of VEGF, angiogenesis was still dependent on CAFs [59]. Such results suggest CAFs are an integral part of tumor-induced neovascularization.

### 4.4. Hepatocellular Carcinoma

Hepatocellular carcinoma (HCC) patients with high CAF gene signatures have a worse prognosis [60]. CAFs in hepatocellular carcinoma express large amounts of extracellular components, such as collagen, proteoglycans, and fibronectin which further lead to a stiff extracellular matrix [61,62]. Matrix stiffening is considered one of the factors promoting cancer proliferation and invasion [63]. Schrader et al. found that matrix stiffness promoted proliferation and cisplatin and 5-FU resistance [64]. This was maintained via HGF-induced signaling responses. HGF was also found to promote cancer metastasis and stemness in hepatocellular carcinoma [61,64,65]. Zhang et al. used different hepatocellular carcinoma xenograft models and found that miR-320a up-regulation is correlated with a less aggressive cancer phenotype. Unlike many other miRNAs, miR-320a was down-regulated in CAF-derived exosomes. The authors found that miR-320a can inhibit MAPK signaling activity leading to inhibition of metastasis drivers and EMT [66]. Yang et al. found that CAFs were crucial for the recruitment of MDSCs through expressing CCL2, which led to MDSCs recruitment in a CCR2-dependent manner [67]. CAFs also play an important role in the recruiting and polarization of macrophages [68]. An in vivo study found that the interaction between endosialin on surface CAFs and CD68 on macrophages leads to more macrophage recruitment and polarization to M2 cells [69]. Inhibition of endosialin leads to decreased M2 infiltration.

### 4.5. Cholangiocarcinoma

The concept of CAFs is poorly studied and characterized in liver tumors, particularly in the highly desmoplastic cholangiocarcinoma. A single-cell analysis conducted by Affo et al. explores CAF functions in intrahepatic cholangiocarcinoma (ICC). It identifies hepatic stellate cells (HSCs) as the primary source of CAF, showing that CAF promotes ICC progression in mice and correlates with poor patient outcomes. Single-cell RNA sequencing distinguishes CAF subpopulations (iCAF and myCAF) with unique interactions. myCAF promotes ICC via hyaluronan synthase 2, while iCAF enhances ICC growth through HGF-MET signaling, offering potential therapeutic targets. Type I collagen does not play a significant role in ICC growth [70]. Another study was carried out by Lan et al. on the effect of Caveolin-1 (CAV1)-positive CAFs on survival outcomes and tumor progression [71]. They reported that up-regulated CAV1 in CAFs was associated with poor overall survival and that was positively related to advanced stage, lymph node involvement, and forkhead box protein 3-positive (FoxP3+) tumor-infiltrating lymphocytes (TILs) [71,72]. No correlation between CAV1 expression and CD8+ TILs was observed. CAV1 plays a role in cellular senescence, and its expression results in the secretion of tumor-stimulatory factors such as IL-6. Thus, the expression of CAV1, which is a marker of CAF’s senescence, plays a crucial role in determining the prognosis of ICC patients through regulating Foxp3+ TILs and hence provides a potential therapeutic target [71].

### 4.6. Colorectal

High CAF infiltration in the TME is associated with a more aggressive disease biology and a worse prognosis in CRC [73]. Zhang et al. described the role of CAFs in attracting monocytes via secretion of IL-8 followed by promoting their polarization to M2 immune-suppressor cells with resultant inhibition of the NK cell activity in CRC [74]. Herrera et al. found that the presence of synchronous CAFs and M2 signatures results in a worse prognosis in CRC [75]. Collectively, these findings suggest that both M2 and CAFs work synergistically in promoting an immunosuppressive TME and associated worse prognosis. In addition to FAP being one of the biomarkers of CAFs, patients with a hereditary mutation of it are at 100% risk of developing CRC [76]. CD70 has been a center of attention in both hematological and solid cancers. Although it is considered a co-stimulatory molecule when binding to CD27, it has been found to facilitate immune evasion and tumor progression in hematological cancers [77]. In solid tumors, CD70 does not bind to CD27 but, still, it leads to EMT and a more metastatic phenotype [78]. Interestingly, the expression of CD70, a co-stimulatory molecule, on CAFs is associated with more invasive CRC [77]. Ren et al. found that CAFs promote stemness and oxaliplatin resistance in a colitis-associated cancer mouse model by transferring exosomal H19 to the stroma [79]. In addition, CAFs induce miR-24-3p secretion, which has been associated with chemoresistance to anti-metabolites in CRC models [80,81]. Targeting miR-24-3p has led to the restoration of chemosensitivity in these models. Regarding targeted therapies, CAFs have been associated with resistance to cetuximab, an EGFR monoclonal antibody, through inducing EGF section by CAFs [80]. Such evidence enlightens the high potential therapeutic role of targeting CAFs while using standard FDA-approved treatments.

## 5. Understanding CAF Subsets Using Single-Cell Transcriptomics

Single-cell RNA sequencing (scRNA-seq) has been used to determine the heterogeneity in tumors on the cellular and molecular levels [82]. It can also be used to unveil malignant and non-malignant cells within tumors [83,84]. However, this has been challenging in certain types of tumors such as pancreatic ductal adenocarcinoma (PDAC). In a recent study by Hwang et al., they built a detailed molecular map of the cellular subtypes and spatial communities that constitute PDAC using scRNA-seq and whole-transcriptome digital spatial profiling (DSP) techniques. Their study highlighted inter- and intra-tumoral heterogeneity and the spatial organization of cell lines in distinct communities as well as revealing the impact of treatment on tumor cells, which in turn provided clinically important information regarding prognosis [85].

The capability of scRNA-seq in marking the TME’s heterogeneity and diversity has shed light on drug resistance mechanisms and discovered more effective therapeutic targets [86]. A critical role of CAFs was demonstrated in a study conducted by Li et al. in which they used scRNA-seq primarily to reveal the categorization and roles of distinct CAF subsets in gastric cancer (GC). They reported that CAFs have a crucial role in regulating various aspects of the TME, such as immune modulation, invasion, migration, and angiogenesis. Particularly, they demonstrated that activated CAFs (eCAFs) exhibit an increased chemotaxis capability in attracting M2 macrophages and are linked to a negative prognosis in GC patients. These results highlight the possibility of targeting eCAF activation as a potential therapeutic strategy for individuals with GC [87].

On the other hand, in HCC, the secretions of CAF and CAF gene signatures have been strongly associated with poor prognosis [88]. A supporting study that was carried out on HCC used scRNA analysis, and they identified three clusters of CAFs that showed a substantial correlation with HCC prognosis [89]. These clusters were distinguished using a score based on the Differentially Expressed Genes (DEGs) found across all four clusters. Notably, they also observed variations in the expression of HIPPO and MYC among the CAF clusters, which could potentially play a role in influencing the prognostic significance of CAFs [90]. It is worth mentioning that Hepatic Hippo signaling has been linked to inhibiting the development of HCC, and the MYC-mediated axis has been widely recognized as a critical component in HCC, impacting proliferation, migration, invasion, and drug resistance [91].

## 6. The Dynamic Interplay between CAFs and the Immune System

**Figure 1:**
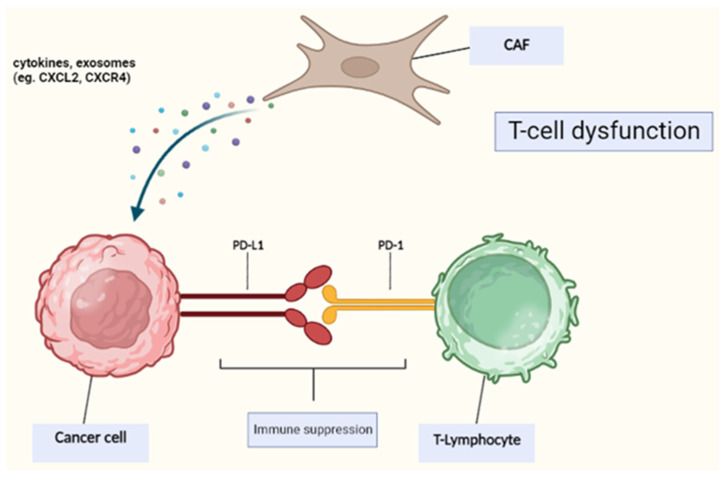
CAFs secrete molecules inducing PD-L1 expression on tumor cells, which binds to PD-1 on T-cells, leading to T-cell dysfunction.

The interactions between CAFs and immune cells in the TME drive an immunosuppressive effect, which in turn enables tumor cells to bypass the surveillance of the immune system [92]. In addition, CAFs interact with the immune checkpoint molecules that are expressed on the cell surface to induce immunologic tolerance by upregulating their gene expression. The high expression of these molecules on T-cell surfaces and tumor cells has been shown to contribute to T-cell dysfunction in the TME [93]. Moreover, PD-1 and PD-L1 are critical immune checkpoint molecules, and their binding on activated T-cells hinders antitumor effects by counteracting the signals of activated T-cells [94]. In addition to upregulating molecules on their surface, cancer-associated fibroblasts (CAFs) also generate a diverse array of cytokines and exosomes that lead to the upregulation of checkpoint molecules on neighboring cells, including tumor cells and immune cells within the TME. This process indirectly results in the inhibition of T-cell function and antitumor responses. In summary, CAFs have a dual role in the TME: not only do they upregulate and trigger the expression of the immune checkpoint molecules, but they also induce the endogenous overexpression of their corresponding legends, hence contributing to the dysfunction of infiltrating tumor T-lymphocytes and promoting immunologic tolerance within the TME. Further work is needed to deeply understand the pathophysiology of the interaction between CAFs and the immune system to establish CAF-targeted immunotherapies (Figure 1). Several studies have also demonstrated that the interaction between cancer cells and CAFs upregulates PD-L1 expression, ultimately increasing tumor growth and progression, especially in CAF-rich tumors such as esophageal cancer [95,96]. In this case, anti-PD-L1 antibodies can be used to suppress tumor growth. However, in other cases such as non-small cell lung cancer or triple-negative breast cancer, the expression of PD-L1 by CAFs was associated with better outcomes [97,98]. Hence, we conclude that the effect of PD-L1 expression by CAFs on survival outcomes depends mainly on both cancer and histological types. In addition, the response to anti-PD-L1 varies depending on the proportion of CAFs expressing PD-L1 [95].

## 7. CAF-Targeted Therapy and Immunotherapy Approaches

**Figure 2:**
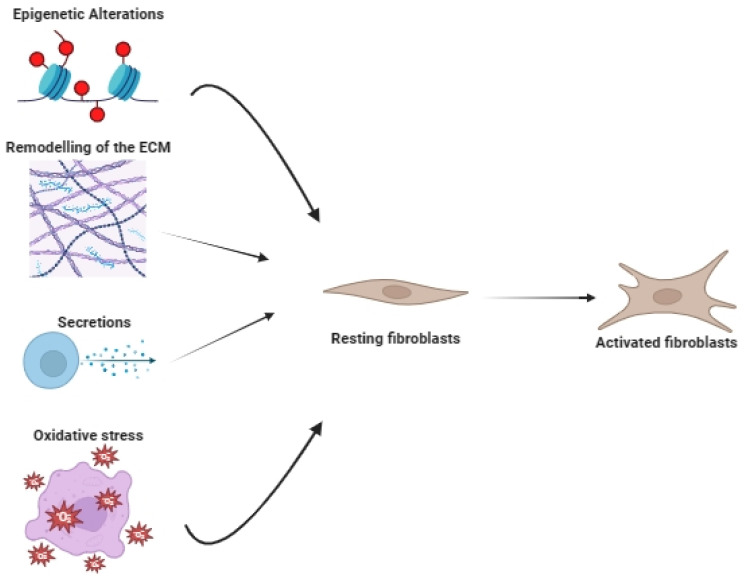
The activation process of CAFs.

CAFs have become an emerging target for therapeutic interventions within the tumor microenvironment due to their significant role in promoting tumor growth and metastatic spread. Numerous studies have emphasized the necessity of simultaneously targeting tumor and stromal cells to eliminate large solid tumors effectively. These studies have shown that eliminating tumor stroma enables the eradication of tumor cells that have downregulated the targeted antigen with a mechanism independent of antigen expression [99,100]. CAFs can trigger the rapid proliferation of cancer cells with the release of growth factors, which cause cells to outgrow the existing vasculature. As a result, hypoxia develops in these regions, reducing the efficacy of treatments that rely on oxygen or free radicals. To achieve a lasting therapeutic response, it is essential to target not only tumor cells but also stromal elements like CAFs. This idea has gained support from immunotherapeutic studies, demonstrating that effectively eradicating large solid tumors relies on simultaneously targeting both cancer cells and stromal elements [101,102]. Figure 2 shows the activation process of CAFs.

Of the growth factors produced by CAFs are the Platelet-Derived Growth Factor (PDGF) and its receptor (PDGFR), which play a vital role in controlling cell growth and survival. However, when PDGF becomes overactive as a result of overexpression or mutations, it can lead to uncontrolled cell growth and ultimately tumorigenesis [103]. This hyperactivity of PDGFR led to research efforts exploring the use of PDGF and PDGFR antagonists as a potential treatment strategy in various cancers [104]. Imatinib, a tyrosine kinase inhibitor (TKI) targeting primarily PDGFR, was approved by the FDA in 2012 as an adjuvant treatment of patients with Kit+ (CD117+) gastrointestinal stromal tumors (GISTs) after complete resection, following the results of a phase III multicenter randomized open-label trial [105]. In clinical settings, Imatinib and other TKIs targeting PDGFR and Kit have been regularly used and have shown favorable outcomes [106,107,108]. The optimal approach, whether with selective inhibition using monoclonal antibodies or ligand traps, or less specific inhibition with low-molecular-weight inhibitors, is yet to be determined and requires further investigation for achieving the best clinical outcomes [109,110].

Recent clinical trials have shown a new potential cancer treatment method called near-infrared photoimmunotherapy (NIR-PIT), which is a method that uses an antibody-photo-absorber conjugate (APC) consisting of a specific monoclonal antibody linked to a photoactivatable phthalocyanine-derived dye [111]. NIR-PIT directed at CAFs effectively suppressed tumor progression in co-culture models of esophageal cancer and CAFs [96,112]. A way by which NIR-PIT works is to target FAP that is specifically expressed in CAFs. In this way, NIR-PIT can overcome the resistance possessed by CAFs in esophageal cancer, as reported by Watanabe et al. [113]. Consequently, the treatment of esophageal cancer may benefit from a combination approach involving targeting EGFR, HER2, or CAFs with NIR-PIT [114]. In the future, it may be feasible to use combinations of APCs to address specific cancer types more comprehensively [111].

## 8. Conclusions

CAFs play a pivotal role in the modulation of TME. Targeting these cells and their secretory products may provide a potential target for reducing tumor-promoting events by blocking metastasis, proliferation, and migration of tumor cells. Developing CAF-targeted therapies alongside immunotherapy may be the next evolutionary step in the eradication of cancer. However, several challenges exist such as the heterogeneity in CAFs in the TME, the multifaced roles across different tumor types, and the lack of in vivo studies that illustrate the molecular and biological roles of CAFs.

## 9. Future Directions

Emerging therapies require an understanding of cancer at all levels including the molecular and cellular levels. Developing a solid background of each cancer hallmark will facilitate the development of tailored targeted therapies with reduced toxicities and adverse effects. CAFs are emergent structures that play a vital role in the evolution of tumorigenesis and TME. Different mechanisms and roles of CAFs occur in different tumors, and to understand the pathophysiology of how each subtype works, we need to investigate their role across each tumor with more in vivo work to better characterize and identify their function. Single-cell spatial transcriptomic analyses are encouraged for genetic studying and mapping, which could offer valuable insights into the architecture, hierarchy, and plasticity of CAFs.

## Data Availability

No new data were created or analyzed in this study. Data sharing is not applicable to this article.
